# Phytochemicals Targeting BDNF Signaling for Treating Neurological Disorders

**DOI:** 10.3390/brainsci15030252

**Published:** 2025-02-27

**Authors:** Alka Ashok Singh, Shweta Katiyar, Minseok Song

**Affiliations:** 1 Department of Life Sciences, Yeungnam University, Gyeongsan 38541, Republic of Korea; alkasingh10f@gmail.com; 2 Department of Botany, SBN Government PG College, Barwani 451551, MP, India; shwetakatiyar@gmail.com

**Keywords:** neurological disorders, neurotrophins, BDNF signaling, phytochemicals

## Abstract

Neurological disorders are defined by a deterioration or disruption of the nervous system’s structure and function. These diseases, which include multiple sclerosis, Alzheimer’s disease, Parkinson’s disease, Huntington’s disease, and schizophrenia, are caused by intricate pathological processes that include excitotoxicity, neuroinflammation, oxidative stress, genetic mutations, and compromised neurotrophic signaling. Although current pharmaceutical treatments relieve symptoms, their long-term efficacy is limited due to adverse side effects and weak neuroprotective properties. However, when combined with other neuroprotective drugs or adjunct therapy, they may offer additional benefits and improve treatment outcomes. Phytochemicals have emerged as attractive therapeutic agents due to their ability to regulate essential neurotrophic pathways, especially the brain-derived neurotrophic factor (BDNF) signaling cascade. BDNF is an important target for neurodegenerative disease (ND) treatment since it regulates neuronal survival, synaptic plasticity, neurogenesis, and neuroprotection. This review emphasizes the molecular pathways through which various phytochemicals—such as flavonoids, terpenoids, alkaloids, and phenolic compounds—stimulate BDNF expression and modulate its downstream signaling pathways, including GSK-3β, MAPK/ERK, PI3K/Akt/mTOR, CREB, and Wnt/β-catenin. This paper also highlights how phytochemical combinations may interact to enhance BDNF activity, offering new therapeutic options for ND treatment. Despite their potential for neuroprotection, phytochemicals face challenges related to pharmacokinetics, blood–brain barrier (BBB) permeability, and absorption, highlighting the need for further research into combination therapies and improved formulations. Clinical assessment and mechanistic understanding of BDNF-targeted phytotherapy should be the main goals of future studies. The therapeutic efficacy of natural compounds in regulating neurotrophic signaling is highlighted in this review, providing a viable approach to the prevention and treatment of NDs.

## 1. Introduction

Neurological disorders (NDs) are the second leading cause of death and the primary cause of disability worldwide [1]. The global population is aging, leading to a growing social and health burden. As neurological diseases become more prevalent, their progressive nature makes them incurable. Current treatments only help alleviate symptoms or slow disease progression [2]. NDs encompass a range of diseases that affect the entire nervous system, including the peripheral nervous system (PNS) and central nervous system (CNS) [3]. Neuroprotection is aims to prevent neuronal death, restore the neural network and alleviate brain dysfunction as “disease-modifying” therapies for depression, Parkinson’s disease (PD), and Alzheimer’s disease (AD) [4].

NDs are caused by a wide variety of mechanisms, including programmed cell death, aggregated proteins, oxidative stress, excitotoxicity, and aging [5,6,7]. The ratio of antiapoptotic (e.g., Bcl-2, Bcl-xL) to proapoptotic (e.g., Bax and Bak) Bcl-2 family proteins must remain balanced for proper cellular function. An imbalance between antiapoptotic and proapoptotic members of the Bcl-2 protein family leads to neuronal damage and contributes to NDs [8,9]. Furthermore, the extrinsic pathway interacts with the intrinsic apoptotic system through the caspase-dependent activation of the Bid protein, leading to the amplification of apoptosis [3]. The brain-derived neurotrophic factor (BDNF) promotes cell survival by activating the ERK1/2 and PI3K/Akt signaling pathways. One of the most significant findings of this signaling is the regulation of Bcl-2 family proteins, which comprise both anti-apoptotic (Bcl-2, Bcl-xL) and pro-apoptotic proteins (such as Bax, Bak, Bid, Bad, and Bim) [10]. BDNF signaling specifically increases the expression of anti-apoptotic proteins such as Bcl-2 and Bcl-xL, which promotes neuronal survival by inhibiting apoptosis. Furthermore, decreased neuronal density and shrinkage in sections of the bipolar brain, such as the frontal and subcortical regions, may be due to an increase in apoptotic activity [11]. Studies consistently demonstrate that altered BDNF communication pathways are related to the development and progression of neurological conditions, highlighting their potential as therapeutic targets [12].

Neurotrophins, a protein family that includes NGF, NT-3, NT-4/5, and BDNF, are essential for neuronal survival, maintenance, and function. Decreased neurotrophin levels have been linked to a variety of neurodegenerative diseases [13,14,15,16,17,18,19,20,21,22]. Neurotrophins have been explored as therapeutic targets for neurodegenerative diseases, with strategies including gene therapy, small-molecule mimetics, peptidomimetics, and nanoparticle-based delivery systems to enhance their stability and blood–brain barrier permeability [23,24,25]. BDNF belongs to the neurotrophin family of growth factors. It is critical to the survival and function of striatal neurons. Depletion of BDNF has been associated with striatal neuron degeneration and death, which results in Huntington’s disease-like motor, cognitive, and behavioral dysfunctions [26]. Throughout development and aging, BDNF, a crucial neurochemical engaged in multiple growth and maturation phases, regulates synaptic plasticity and strengthens the endurance and specialization of neurons [27]. Neurotrophic factors are signaling molecules that help cells communicate across the nervous system and play important roles in neuronal growth, survival, and functional adaptability. Insulin-like growth factor-1 (IGF-1), GDNF, and BDNF are examples of chemicals produced by neurons, glial cells, and even peripheral organs that operate as modulators in a network of cellular interactions [28,29]. Neurotrophins and their receptors, such as the Trk receptors (A, B, and C) and the p75 receptor (a nonenzymatic member of the TNFR family), are essential for the survival of neurons. TrkB activation by BDNF or NT-4/5 promotes synaptic function, neuronal survival, and differentiation. Neurodegenerative illnesses like Huntington’s, Parkinson’s, and Alzheimer’s are associated with disruption of the BDNF/TrkB signaling system [30].

Given the significance of BDNF in neuronal function and disease pathology, it remains important to find safe and effective approaches to modify its signaling pathway. Phytochemicals, or bioactive compounds produced from plants, have received attention for their neuroprotective qualities, which include the ability to modulate BDNF expression and function.

However, natural phytochemicals are often considered less toxic than newly developed synthetic drugs. Nevertheless, traditional herbal medicines, typically derived from crude plant materials, pose several challenges, including uncertainties regarding their precise medicinal effects, consistency in therapeutic outcomes, mechanisms of action, and identification of active compounds [31]. The purpose of this study is to investigate the potential of phytochemicals in targeting BDNF signaling for the treatment of NDs. This includes identifying important phytochemicals that can upregulate or modify BDNF pathways, determining their mechanisms of action, and assessing their therapeutic efficacy.

Understanding the critical role of BDNF in the pathology of neurological diseases lays the groundwork for creating novel therapeutic techniques. By investigating phytochemicals’ potential to influence BDNF signaling, researchers can discover alternate routes for improving existing drugs or perhaps treating previously incurable diseases. This study intends to bridge the gap between experimental findings and clinical applications by providing insights into how phytochemical-based therapies can help with neuroprotection and disease modification in diverse NDs.

## 2. Search Methodology

To offer a thorough overview, we conducted a systematic literature search in PubMed and Google Scholar. Only peer-reviewed English-language published articles were considered. Relevant studies were identified using keywords such as ‘BDNF and neurodegeneration’, ‘BDNF signaling in Alzheimer’s disease’, and ‘phytochemicals targeting BDNF’, as well as ‘BDNF and neurodegeneration’, ‘Phytochemicals targeting BDNF’, ‘Terpenoids and neuroprotection’, ‘Neuroprotective effects of phytochemicals’ and ‘Neuroprotective therapies targeting BDNF’. The selected articles were extensively analyzed in order to summarize current findings and highlight emerging therapy approaches. However, in our review, we have selected only those compounds that exhibit the most potent therapeutic effects in the treatment and management of neurological diseases by modulating BDNF signaling, based on evidence from both in vitro and in vivo studies.

## 3. Brain-Derived Neurotrophic Factor (BDNF)

**Figure 1:**
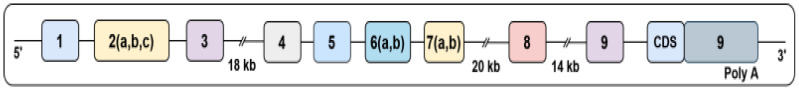
Intron and exon arrangement of the human BDNF gene; alternative polyadenylation sites (PolyA) in the 3′-UTR and internal alternative splice sites in exons 2, 6, and 9 (letters a, b, c, and d) are indicated by the structure and splicing variation in human BDNF (created by using draw.io, a free standalone application version: v26.0.16).

Brain-derived neurotrophic factor (BDNF) is a factor that supports the proliferation [32], maturation [33], and existence of neurons in the nervous system [34]. It is responsible for neurogenesis and neuroprotection during neurotoxicity. There are eight different mRNAs transcribed in human BDNF; transcripts with exons I through III occur primarily in the neural network, whereas exon IV is located in the heart and lungs. According to the results of the in situ hybridization experiment, BDNF mRNA is highly expressed in the brain. Numerous genes have been identified whose expression is triggered by various neural plasticity-generating pathways; however, gene expression level changes constitute only a small part of the activity-regulated transcriptional program. Alternative splicing of precursor mRNA is another method that influences the activity-dependent transcriptional profile [35]. Additional research found that the activity-dependent elevation of BDNF transcripts from exon IV is independent of translation activity, whereas the induction of transcripts from exons I and II was susceptible to protein synthesis inhibition [36] (Figure 1). The BDNF gene produces several transcripts with different 5′- and 3′-untranslated region (UTR) segments, which have diverse subcellular locations. In rats, there are 22 BDNF transcripts that code for the same protein. The 11 separate 5′-UTRs are alternatively spliced into a common downstream exon, exon 9, which comprises the coding sequence, as well as a 3′-UTR with two polyadenylation sites [37]. The CDS (coding sequence) refers to the section of a gene that is transcribed into mRNA and translated into a functional protein. The CDS of the BDNF gene is largely found in exon 9, which encodes the full-length BDNF protein, but exons 1–8 contribute to transcript variety by influencing translation efficiency via various 5′UTR sequences [38]. Furthermore, immune cells also expressed BDNF in inflammatory central nervous system lesions in experimental autoimmune encephalomyelitis (EAE) models and patients with multiple sclerosis. This led to the concept that BDNF could mediate immune cells’ neuroprotective effects [39]. Low levels of BDNF activity are present during fetal development but rise significantly after birth and then decline in adults [40].

## 4. BDNF Signaling Mechanism

**Figure 2:**
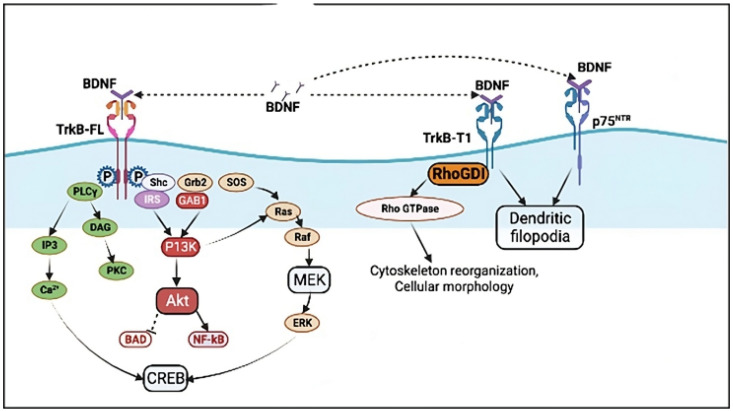
BDNF/TrkB signaling pathway: three main signaling pathways—MAPK/ERK, PI3K, and PLCγ—are activated when BDNF binds to TrkB-FL in neurons, leading to receptor homodimerization and stimulation and regulating various processes essential to neuronal function. As an alternative, TrkB-T1 also plays a role in regulating the level of BDNF in a given area as well as the shape of individual cells in astrocytes and neurons (created by draw.io, version v26.0.16).

Brain-derived neurotrophic factor (BDNF), one of the most common neurotrophic factors in the central nervous system (CNS), contributes significantly to CNS injury by attaching to its unique receptor, Tropomyosin-related kinase receptor B (TrkB). The BDNF/TrkB signaling system is critical for neuronal survival, remodeling, and plasticity [41]. Depression is frequently accompanied by decreased BDNF levels and compromised TrkB signaling, which lead to neuronal atrophy, synaptic dysfunction, and decreased neuroplasticity [42]. BDNF binds with high affinity to TrkB and with low affinity to p75^NTR^. In the human brain, the TrkB receptor is expressed in four isoforms: TrkB-FL (full length), TrkB-T1 (truncated), TrkB-Shc (Src-homology 2-domain containing adaptor protein) (lacking a tyrosine kinase domain), and TrkB-T-TK (having a non-functional catalytic domain) [43]. The actions of ProBDNF and mBDNF’s depend on their ability to bind to the TrkB and p75 neurotrophin receptor (p75NTR) receptors, respectively [44]. Studies show that the proBDNF/p75NTR/sortilin complex stimulates signaling pathways involving c-jun N-terminal kinase, Ras homology gene family member A, and nuclear factor κB. This activation reduces neurogenesis, synaptic development, and neuronal survival while increasing neuronal apoptosis [45]. BDNF initially forms in the endoplasmic reticulum as a precursor, pro-BDNF, which is a physiologically active factor that differs from mature BDNF. Pro-BDNF can impair dendritic complexity and synaptic plasticity in the hippocampus, but mature BDNF has an opposite effect in the CNS [46,47]. BDNF binding with TrkB-FL induces receptor dimerization and autophosphorylation. Upon the phosphorylation of RTKs, multiple signaling cascades are activated, each regulating specific cellular functions. The mitogen-activated protein kinase (MAPK) pathway, which regulates gene expression and orchestrates cellular processes such as proliferation and differentiation, is particularly active. Concurrently, the Phosphoinositide 3-Kinase/Protein Kinase B (PI3K/Akt) pathway is activated, which controls several aspects of cell survival, growth, and metabolism [48]. The PLCγ (Phospholipase C γ) pathway is another important pathway that has been activated. It plays a significant role in regulating calcium signaling and cytoskeletal reorganizations [49]. Studies on neurodegeneration, such as 3-nitropropionic acid (3-NP)-induced models, have highlighted the critical role of PLCγ in NT-3-mediated plasticity in striatal tissue, especially in neurodegenerative diseases like 3-NP. The recovery of striatal LTD (long-term depression) is prevented by the inhibition of PLC, but not of PI3K or MEK/ERK, indicating that PLCγ signaling is essential for NT-3 neuroprotection. Furthermore, endocannabinoid signaling is modulated by PLCγ, and its role in NT-3-mediated plasticity is evidenced by CB1 receptor inhibition using AM251. This suggests that PLCγ could be a target for the development of pathway-specific medications for neurodegenerative illnesses [50]. TrkB receptor activation by BDNF binding initiates three major, intricately linked signal transduction cascades, MAPK/ERK, PI3K, and PLCγ (Phospholipase C γ), which regulate several neuronal functions (Figure 2). Tyr515 phosphorylation allows for the docking of Shc to the receptor to initiate the PI3K/Akt pathway [51], which regulates the activity of various proteins necessary for neuron existence, such as BAD (Bcl-2 antagonist of cell death) and GSK-3 (Glycogen Synthase Kinase 3) [52]. Shc also activates the MAPK/ERK pathway, which requires the attachment of FRS2 (adaptor protein) at the Tyr515 residue or the activity of the TrkB-interacting protein Kidins220 (Kinase D-interacting substrate of 220 kDa). Activation of PLCγ by Tyr816 phosphorylation is also associated with neurite development and synaptic plasticity [53]. The other main isoform, TrkB-T1, interacts with Rho GDI (Rho GDP dissociation inhibitor 1) to influence cell shape and modulate local BDNF levels [54].

## 5. Cross-Talk Between BDNF and Other Signaling Pathways

BDNF plays a significant role in neuronal growth [26], plasticity [55], and survival by interacting with multiple intracellular signaling pathways [56]. This chapter explores how BDNF interacts with major signaling cascades such as Wnt/β-catenin, CREB, GSK3, and mTOR, with a focus on its impact on neurodevelopmental and neuropsychiatric disorders.

### 5.1. BDNF and Wnt/β-Catenin Signaling Pathways

MAPK and BDNF are two critical participants in cellular signaling, and their dysregulation has been linked to a variety of neuropsychiatric conditions. BDNF has been shown in studies to improve schizophrenia (SCZ) behavior by controlling nervous system building, influencing the growth and distribution of neurons and glial cells, and altering inflammation and apoptosis in the brain [57]. The study examined MAPK- and cAMP-dependent signaling pathways in the ACC and DLPFC in schizophrenia. In the ACC, Rap2, JNK1/2, pT183/Y185 JNK1/2, and pS295 PSD-95 expression was reduced, impacting MAPK signaling. Cdk5, Rack1, Fyn, pS295-PSD-95, and pY1336-NR2B expression levels were found to be elevated in the DLPFC, affecting cAMP signaling. These proteins are critical for neurotransmission integration, and dysregulation may lead to broad signaling abnormalities in schizophrenia. These findings imply that schizophrenia has a link with aberrant activity of the cAMP- and MAPK-associated pathways in frontal cortical regions [58]. Cross-talk across these pathways has been discovered to play a crucial role in a variety of physiological and pathologic disorders. Mitogen and stress-activated kinases (MSKs) 1 and 2 are nuclear proteins that activate in response to ERK1/2 or p38 MAPK signaling [59]. The research discovered that BDNF promotes neural stem cell (NSC) proliferation and differentiation, potentially through the Wnt/β-catenin signaling pathway. First, BDNF enhanced the proliferation and differentiation of NSC cells in a dose-dependent manner [60]. BDNF and Wnt signaling are interesting therapeutic targets because they converge on important neuroplasticity pathways implicated in depression. Wnt proteins, including Wnt2, Wnt3a, Wnt5, and Wnt7a/b, interact with BDNF signaling to promote synaptic plasticity, particularly in the hippocampus. A downstream Wnt effector, GSK-3β, may be inhibited to enhance antidepressant effects. Ketamine’s rapid-acting effects are also influenced by the mTOR pathway, which overlaps with BDNF and Wnt signaling. This suggests that combinatorial techniques that alter these interrelated pathways may increase the effectiveness of antidepressants [61]. BDNF activated the Wnt/β-catenin signaling pathway, leading to increased levels of free β-catenin and Wnt1. The Wnt signaling blocker IWR1 drastically reduced Wnt/β-catenin signaling, as well as NSC proliferation and differentiation [62]. BDNF also enhanced proliferation and migration in human myeloma cells by activating the MEK/ERK and PI3K/AKT signaling pathways [63], and a shift in the ERK/p38 MAPK activity ratios affected chondrocyte differentiation [64]. In addition, receptor tyrosine kinases may activate Wnt/β-catenin signaling via regulating MAPK/LRP6 and β-catenin phosphorylation [65]. The MAPK/ERK and Wnt/β-catenin pathways worked together to promote the proliferation of Sca-1-positive hepatic progenitors [66]. Cross-talk between Wnt/β-catenin and PI3K/AKT signaling promotes hematopoietic stem cell renewal and expansion [67]. The PI3K signaling pathway activates β-catenin during the host’s defense response to infection [68]. However, the PI3K/AKT or STAT3 pathways may potentially induce Wnt/β-catenin signaling cascades. The MAPK/ERK signaling pathway may potentially play a role in this cross-talk [69]. Additionally, the interaction between BDNF and other signaling pathways, such as Wnt/β-catenin, suggests a promising avenue for the development of treatment strategies in neurological diseases [70]. BDNF signaling has shown strong neuroprotective and regenerative benefits, particularly in dopamine neurons, via both endogenous mechanisms and therapeutic approaches. Combining the BDNF and Wnt/β-catenin pathways may improve neuroprotection, cell transplantation, and gene treatments by enhancing neuronal survival, plasticity, and repair. Targeting this relationship provides a fresh approach to finding successful treatments for disorders such as Parkinson’s and Alzheimer’s [62,71,72,73,74].

### 5.2. BDNF and the CREB Signaling Pathway

The relationship between CREB signaling and BDNF is vital because CREB activation is linked to BDNF’s neuroprotective and prosurvival behaviors [75]. BDNF is a critical neurotrophin that promotes neuronal cell formation and provides neuroprotection in a variety of circumstances [76]. According to research, exercise can improve cognitive performance in people with Parkinson’s disease. Exercise encourages neuroplasticity and the formation of new neurons, which can aid in cognitive function. Exercise has been shown to increase BDNF levels, which are crucial for neuronal survival, growth, and differentiation [77]. The development of long-term versus short-term memory is determined by the duration of BDNF signaling, which also has a significant impact on neuronal survival and CREB phosphorylation kinetics. Transient CREB phosphorylation driven by short-term BDNF signaling is adequate to activate immediate early genes (IEGs) such as c-Fos and Egr-1. Nevertheless, the transcriptional alterations necessary for long-term memory are not maintained by this brief stimulation [78]. The structural and functional alterations required for long-term memory are not triggered by transient CREB activation, although it does support short-term synaptic changes like short-term potentiation (STP) [79]. Long-term memory consolidation requires persistent CREB phosphorylation and prolonged BDNF signaling, which in turn drive structural alterations at synapses as well as the production of new proteins [80]. Impaired neural activity, such as growth and synaptic transmission, can lead to anxiety or depression when the signaling pathway malfunctions [81]. The interplay between CREB and BDNF is vital for sustaining neuronal integrity. Research suggests that BDNF promotes CREB phosphorylation, establishing a positive feedback loop that enhances BDNF gene transcription, which is crucial for synaptic plasticity and memory formation [82]. BDNF activates CREB by increasing intracellular Ca^2+^ levels, which then activate Ca^2+^-calmodulin-dependent protein kinase IV (CaMKIV) [82]. The dendritic growth is essential for neuronal circuit activity. Among the many neurotrophins that regulate dendritic development, BDNF plays a crucial role in modulating dendritic length and complexity via the MAPK and CREB signaling pathways [83]. In addition, BDNF leads CREB’s activation with the cypin promoter region, a key binding protein for postsynaptic density protein-95 (PSD-95), which increases cypin transcription and, in turn, dendritic branching [84,85]. Upregulation of BDNF mRNA correlates with higher expression of phosphorylated CREB. Disruption of the CREB/PKA pathway leads to lower neuronal BDNF levels [86].

### 5.3. BDNF and GSK3 Signaling Pathway

BDNF, a neurotrophin, and GSK3β, a versatile kinase, are key regulators implicated in depression [87,88,89]. Given their roles in mental illnesses, the convergence of BDNF and the GSK-3β pathways is critical for understanding neuropsychiatric disorders [90,91]. It has been demonstrated that BDNF uses its interaction with TrkB to activate PI3K and Akt, which in turn inhibits GSK-3β activity. However, GSK3 is essential for controlling a wide range of transcription factors and modulators that affect the expression of genes linked to mood regulation and diseases, such as neurotrophins [92,93]. Concurrently increasing BDNF and inhibiting GSK3β together produces a neuroprotective and neurotrophic environment, which supports the aforementioned idea and clarifies the molecular foundation for treating mood disorders [94]. Similarly, it is suggested that in mouse models of depression, the improvement of depression is associated with the modification of the BDNF/TrkB-GSK-3β signaling pathway [95,96]. Pharmacological inhibition of GSK-3β has raised the mRNA levels of BDNF in cortical neuronal cells, which is consistent with the findings above [97]. Overall, the interaction between the GSK-3β and BDNF pathways forms a complex regulatory network essential for neuronal function. From mood modulation to synaptic plasticity, their interaction affects important facets of neuronal function. Deciphering the complexities of this crosstalk is crucial to improving our knowledge of how the brain works and could lead to novel treatment strategies for conditions affecting the central nervous system [59]. Studies show that whereas lower phosphorylated GSK3β correlates with the severity of depression, greater CREB activity and decreased BDNF levels are linked to AD, particularly in the moderate to severe stages of dementia. Enhancing BDNF levels and CREB activation, which are connected to mood management and cognitive function, may be the main goal of therapeutic approaches. For instance, GSK-3β inhibitors may be used in conjunction with CREB activators (like rolipram) and BDNF enhancers (like 7,8-DHF) to promote neuroprotection, slow cognitive decline, and lessen depressive symptoms in AD and depression. Combining these methods may help restore neurotrophic support and synaptic plasticity, providing a viable multi-target treatment approach for these co-occurring illnesses [98].

### 5.4. BDNF and Nrf2 Signaling Cascade

Nrf2 is activated in response to oxidative stress, although it might be impaired or deficient in neurodegenerative disorders. Nuclear Nrf2 levels have been identified to be substantially reduced in the brains of Alzheimer’s patients [99]. In contrast, while Nrf2 nuclear localization is detected in PD patient samples, the response may be insufficient to prevent neuronal death [100]. Additionally, investigations have found that Nrf2 plays a protective effect in neurodegenerative disorders [101,102]. The transcription factor Nrf2 and brain-derived neurotrophic factor (BDNF) play critical roles in depression. However, the molecular pathways between Nrf2 and BDNF in depression are poorly known [103]. A recent study found that seasonal variations in the Nrf2 pathway can influence winter depression-like behaviors [104]. Nrf2 has the ability to bind with the E3 ligase adapter Kelch-like ECH-associated protein (Keap1), which is its primary negative regulator [105,106,107]. Another study suggests that major depressive disorder (MDD) patients’ parietal cortex showed lower levels of Nrf2 and Keap1 expression than the control group, suggesting that decreased Keap1–Nrf2 signaling could be a major factor in the onset of depression [108]. In the learned helplessness (LH) paradigm, rats with LH (susceptible) showed decreased levels of Nrf2 and BDNF in their medial prefrontal cortex (mPFC) and hippocampus compared to control and non-LH (resilient) rats [108,109]. Abnormalities in the brain’s Nrf2 and BDNF crosstalk might function synergistically to cause depression-like symptoms in rodents. According to a study, Nrf2 binds to the promoter of Bdnf and displaces repressors (HDAC2, mSin3a, and MeCP2) to control Bdnf transcription. Through the use of LPS-treated and Nrf2 KO mice, it examined (1) the effects of Nrf2 activation on BDNF and its repressors, (2) Nrf2-mediated Bdnf transcription using luciferase and ChIP assays, (3) Nrf2-BDNF crosstalk in depression-like behaviors, and (4) their roles in resilience vs. susceptibility in mice exposed to chronic social defeat stress (CSDS) [103]. A recent study found that (R)-ketamine’s antidepressant-like effects in Nrf2 KO mice are mediated by BDNF-TrkB signaling [110]. As a result, it can be anticipated that the communication between Keap1-Nrf2 signaling and BDNF-TrkB signaling in the brain plays an important role in depression.

### 5.5. BDNF and mTOR Signaling Pathway

**Figure 3:**
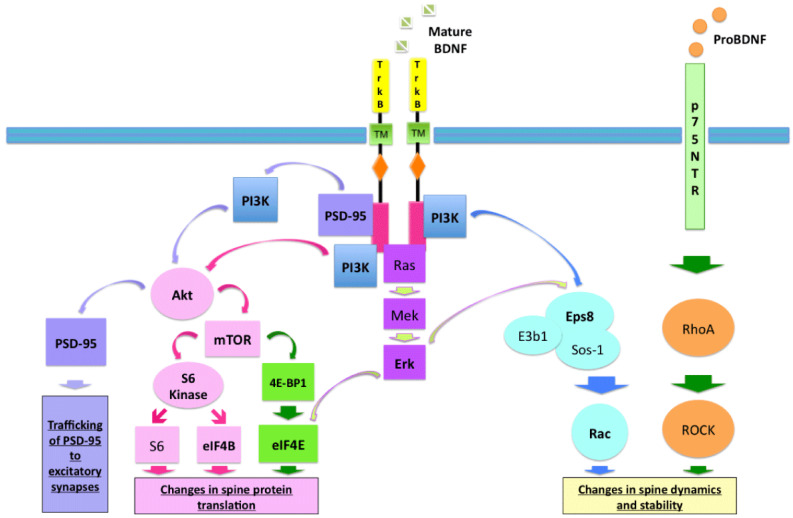
ProBDNF/p75NTR reverses the effects of BDNF/TrkB signaling, which activates the mTOR pathway to control actin remodeling and spine protein production, hence weakening dendritic spines [115]. Copyright: © 2015 by authors Autism Open Access.

A significant “hub” in the regulation of synaptic protein synthesis and neuronal survival is mTOR, and either excessive or insufficient signaling via this route is associated with autism, not necessarily causative. For instance, mutations in TSC1/2 and PTEN lead to hyperactivation of the PI3K-Akt-mTOR pathway, contributing to conditions such as tuberous sclerosis complex and macrocephaly. Individuals with conditions have a higher prevalence of autism compared to the general population. [111,112]. BDNF binding to TrkB-FL activates several intracellular pathways, including PI3K and ERK, which regulate neuronal development and function [113,114]. BDNF/TrkB/PI3K signaling regulates actin remodeling at synapses and spine protein synthesis. These include the Eps8-Rac route, which modulates Rac-dependent actin cytoskeletal remodeling, and the Akt-mTOR pathway, which controls the production of spine proteins through two downstream signaling cascades, 4EBP1/eIF4E and S6 kinase/S6. Additionally, TrkB signaling via PI3K attracts PSD-95 to spines, while Erk signaling phosphorylates eIF4E and Eps8 to contribute to the BDNF-dependent regulation of translation and cytoskeletal reorganization, respectively. Finally, proBDNF/p75NTR destabilizes dendritic spines and counteracts Eps8/Rac signaling (Figure 3) [115,116]. Interestingly, mTOR inhibitors have been shown to reverse autism-like behaviors in adult mouse models [117,118]. Studies show that idiopathic autism is characterized by reduced TrkB signaling through the PI3K/Akt/mTOR pathway [119] and via the Eps8/Rac pathway [120]. Studies indicate that idiopathic autism is characterized by reduced TrkB signaling through the PI3K/Akt/mTOR and Eps8/Rac pathways, while proBDNF activity remains elevated [121]. Unlike other neurodevelopmental disorders such as tuberous sclerosis, neurofibromatosis type I, and Fragile X syndrome, idiopathic autism is linked to reduced TrkB signaling through the PI3K/Akt/mTOR and Eps8/Rac pathways [115].

## 6. The Role of Phytochemicals in Modulating Epigenetic Crosstalk for Holistic Neuroprotection and the Significant Role of Phytochemicals in NDs

Curcumin can protect neuronal health by reducing ROS levels, activating the Nrf2 pathway, and inhibiting the NF-κB pathway [122]. Nrf2 activation causes the transcription of a wide range of cytoprotective genes, including those encoding enzymes such as glutathione S-transferases (GSTs), NAD(P)H quinone oxidoreductase 1 (NQO1), and heme oxygenase-1 (HO-1). These proteins are vital for neutralizing ROS, detoxifying toxic chemicals, and preserving cellular health [123,124]. Curcumin inhibits NF-κB-driven transcription, thereby reducing inflammation and allowing enhanced Nrf2 activation. Curcumin modulates both Nrf2 and NF-κB pathways, enhancing antioxidant defense while suppressing inflammation, making it a potent neuroprotective agent [125]. A study suggests that curcumin, a phytochemical with anti-inflammatory and antioxidant characteristics, can reduce hypoxia-induced white matter damage (WMI) by modulating the NF-κB and Nrf2 signaling pathways [126]. By mitigating inflammation, oxidative stress, and apoptosis, curcumin counteracts key contributors to WMI pathogenesis. It does this by restoring astrocyte shape, reducing vacuolation, and inhibiting pro-inflammatory cytokines like TNF-α and IL-1 [126].

Resveratrol (RSV) modulates the PI3K/Akt and Wnt pathways while interacting with NF-κB signaling, facilitating crosstalk between autophagy, inflammation, oxidative stress, and metabolism [127]. This intricate interplay highlights resveratrol’s potential as a multi-target therapeutic approach for neurological disorders. Resveratrol, a natural compound found in berries, peanuts, and red grapes, exhibits antioxidant, anticancer, and anti-inflammatory properties [128]. Its potential to alter the PI3K/Akt/mTOR pathway has drawn attention in neurodegenerative diseases. Its influence on mTOR, a downstream effector of Akt, adds to its neuroprotective properties. Beyond its effects on PI3K/Akt/mTOR signaling, resveratrol also influences AMPK activation. Studies indicate that resveratrol increases phosphorylated AMPK levels in the cerebral cortex following MCAO and in primary neurons under glutamate-induced excitotoxic stress [129]. RSV enhances PINK1/Parkin-mediated mitophagy, reducing oxidative stress and apoptosis, thereby preserving mitochondrial integrity [130]. Its anti-inflammatory effects reduce inflammation by blocking critical signaling molecules in the system. Furthermore, RSV’s role in improving mitochondrial function contributes to its neuroprotective properties [128].

Honokiol, a lignan found in various Magnolia species, has been used for centuries in traditional Chinese and Japanese medicine for its anti-inflammatory and neuroprotective properties. Magnolias are extensively dispersed over the world, with the majority of species occurring in East and Southeast Asia [131]. Due to its BBB-crossing abilities, honokiol has been identified as a molecule with many therapeutic functions, including anticancer, antibacterial, anti-inflammatory, antithrombotic, antidepressant, and neuroprotective actions [132]. Honokiol improved outcomes in a rat model of TBI by reducing inflammation and endothelial cell damage. This phytochemical increased vascular endothelial growth factor expression and inhibited pyramidal neuron death [133]. In a 2014 study by Wang et al., honokiol (0.5 or 2 mg/kg) improved motor, sensory, and cognitive recovery in a rat model of TBI by reducing apoptosis and lesion size. The substance reduced neuronal apoptosis and lesion size while also inhibiting cell cycle proteins such as cyclin D1, cyclin-dependent kinase 4, retinoblastoma protein, and E2 promoter binding factor 1 [134]. Honokiol exerts neuroprotective effects by reducing inflammation, promoting neuronal survival via neurotrophin signaling, and modulating metabolic and neurodegenerative pathways through PI3K/Akt and MAPK signaling [131].

Some drawbacks of current ND treatment strategies, which primarily manage symptoms rather than address the underlying causes, include poor blood–brain barrier permeability, drug resistance, adverse side effects, and inadequate drug absorption. Natural chemicals derived from plants offer great potential for treating and preventing NDs, according to certain research [135]. Natural derivatives are reportedly a significant source of bioactive compounds and a crucial source of pharmacological leads. Studies indicate that at least one-third of marketed medications are derived from natural sources. As a result, research continues to explore the therapeutic potential of natural derivatives [136]. Numerous herbal remedies have been used in the treatment of neurodegenerative diseases, including AD and other cognitive disorders. Steroids, fatty acids, alkaloids, glycosides, saponins, terpenes, tannins, and other organic substances are phytochemicals present in herbal products. These chemicals have biological activity and the potential to be used as ND treatments [137]. The neurological protection of certain therapeutic herbs utilized throughout history is summarized in Table 1, and the clinical trials utilizing specific phytochemicals have been summarized in Table 2.

### 6.1. Steroids

Medicinal herbs are highly enriched with steroids, having great potential for neuroprotective effects. Spicatoside A, a steroidal saponin discovered to be present in the *Liriope platyphylla* extract, induces neurite outgrowth in PC12 cells and NGF synthesis in astrocytes via the TrkA receptor-mediated activation of PI3-kinase and ERK1/2, which controls long-term potentiation and neuronal activity [138]. According to a report, spicatoside A has been shown to increase the mRNA concentration of BDNF in mice and aid in the recuperation from perceptual impairment [139]. β-sitosterol and stigmasterol isolated from *Ginkgo biloba* oil provide protection against neurodegeneration by multiple mechanisms [159]. Through the suppression of NF-κB and MAPK pathway-mediated inflammation and apoptosis, they demonstrate protective benefits against Aβ25–35-induced damage [160]. These steroids may also regulate caspase-3/PARP-1 signaling, which prevents mitochondria-mediated apoptosis and the harm brought on by Aβ, as well as the p38/-JNK/-NF-κB pathway, suppressing pro-inflammatory mediators [161]. Additionally, there are some marine sterols that have significant anti-inflammatory properties as well. The octocoral Dendronephthya mucronate yielded two steroids, 5α-pregn-20-en-3β-ol and 5α-cholestan-3,6-dione, which were demonstrated to suppress LPS-induced NO generation in activated RAW264.7 murine macrophage cells. Isolated from Dendronephthya sp., dendronesterones D, another octocoral sterol, prevented inflammation by suppressing the production of COX-2 and iNOS. The potential of marine sterols to prevent neuroinflammation in AD pathogenesis is suggested by their anti-inflammatory properties [162].

### 6.2. Phenols

Flavonoids such as apigenin-8-C-β-digitoxopyranoside, apigenin-8-C-β-boivinopyranoside, and luteolin-8-C-β-boivinopyranoside [163], from *Passiflora edulis* and *P. alata*, have been shown to enhance behavioral exposure in rats [164]. It is well known that apigenin and its metabolites have anti-allergic, antioxidant, and neuroprotective effects [165]. In the APP/PS1 mouse model, subchronic therapy with apigenin reduces β-CTF, BACE, and β-amyloid deposition and improves BDNF expression, increasing memory and synaptic plasticity through the ERK1/2/CREB-mediated inhibition of AD [140]. Similarly, Epigallocatechin-3-gallate (EGCG) extracted from *Camellia sinensis* mitigates NDs in APP/PS1 mice by enhancing CREB and NGF activity by TrkA phosphorylation via c-Raf/ERK1/2-mediated neurological protection [166]. In the hippocampus, expression of Aβ((1–40) and amyloid precursor protein are also reduced as a result of EGCG’s ability to diminish p75/CD, JNK2, and split caspase3 activation and expression [167]. Studies show that *C. sinensis* ingestion increases BDNF expression, activates CREB and Bcl-2 protein levels, and protects against age-related neurodegeneration [168]. Additionally, plant material contains catechin, which increases PSD95, BDNF, and CaMKII levels while decreasing Aβ(1–42) levels via the PKA/CREB process in the hippocampus of cognitively and memory-impaired SAMP8 mice [169]. Phenolic compounds demethoxycurcumin, bisdemethoxycurcumin, and curcumin from the plant *Curcuma longa* are used to treat multiple NDs [170]. Curcumin encourages PC12 cell neurite propagation by causing a PKC/ERK1/2-mediated rise in CREB expression as well as boosting the activity of the neurodifferentiation markers GAP43 and NF-L [171]. In an old mouse model, it has been shown that curcumin controls d-galactose-induced learning and spatial deficits by raising CREB and BDNF levels [172]. Resveratrol (3,5,4′-trihydroxy-trans-stilbene) extracted from the *Vitis vinifera* regulates the apoptosis of the peripheral nervous system by the AMP-activated protein kinase-mediated modulation of BDNF, GDNF, and NGF expression [173]. Additionally, resveratrol firmly activates the ERK1/2/CREB-mediated signaling pathway in astrocytes to increase the production of GDNF and BDNF to enhance the survival and development of neurons [173]. A wide range of *Zingiber officinale* (ginger)-derived phenols, including 6-shogaol, 6-gingerol, 8-gingerol, and 10-gingerol, are used for the treatment of various diseases, of which 6-shogaol has been shown to have a specific potential against AD [174]. 6-Shogaol inhibits H_2_O_2_ oxidative stress-induced neuronal death in astrocytes by enhancing GDNF, BDNF, NGF, Bcl-xL, and Bcl-2 and reducing Bax, ROS, and caspase 3 through ERK1/2-mediated signaling [146]. Additionally, 6-shogaol significantly initiates choline transporter, acetylcholine-transferase, and BDNF expression while suppressing ROS production through the BDNF/TrkB-mediated signaling pathway in H_2_O_2_-treated HT22 hippocampal neuronal cells [147]. Oleuropein, another polyphenolic compound extracted from *Olea europaea*, decreases GSH while increasing NGF and BDNF levels in serum and NGF levels in the olfactory lobe and hypothalamus. It also increases BDNF levels in the olfactory lobe while reducing NGF/BDNF levels in the striatum and hippocampus [148].

### 6.3. Terpenoids

Terpenoids are one of the most wide-ranging phytochemicals, showing neuroprotective action both in vitro and in vivo. The active compound of *Ginkgo biloba*, Ginkgolide B, has been used from the ages to treat a variety of neurological conditions, including cognitive dementia and neurosensory problems. Ginkgolide B raises the expression of BDNF levels in primary hippocampal neuron cells that have received the A25–35 treatment. They reduce the caspase 3, lactate dehydrogenase (LDH), and the K^+^ ion levels [175]. It is an effective component for the therapy of neurological disease since it can also cross the blood–brain barrier, especially in ischemic situations [176]. The use of ginkgolic acids (GAs), secondary metabolites of *Ginkgo biloba*, as SUMOylation inhibitors is a relatively new method in cancer therapy. SUMOylation (Small Ubiquitin-like Modifier Conjugation) is a post-translational modification that is necessary for protein stability and cellular activities, including cancer. GAs impair cancer cell survival pathways by blocking SUMOylation, providing a novel approach to antineoplastic therapy in comparison to traditional chemotherapeutic drugs [177]. Dibenzocyclooctadiene lignans, which are also found in *S. chinensis*, have been demonstrated to potentially have neuroprotective effects on SH-SY5Y human neuroblastoma cells. In particular, it has been found that these compounds suppress 6-hydroxydopamine-induced ROS and stimulate CREB and Nrf2 via PKA and PKB [151].

Catalpol, an active component of *Radix preparata,* exhibited a neuroprotective effect by upregulating BDNF expression, boosting myelination, and reducing neuron programmed cell death. In some experiments on spontaneously hypertensive rats, it has been found that catalpol treatment upregulates several regulatory proteins during prefrontal cortex development [152]. Additionally, p35, cyclin-dependent kinase 5 (CDK5), fibroblast growth factor (FGF) 21, and its receptor (FGFR) 1 are also upregulated in response to catalpol [152]. Numerous pharmacological studies have demonstrated the significant therapeutic effects of catalpol and geniposide, two of the most important iridoids in traditional Chinese medicine, on diseases of the central nervous system. It has also been confirmed that their nervous system activities primarily target the intervention of the development process of AD and PD [178]. The study showed that Catalpol reduces LPS-induced cognitive impairment by inhibiting the NF-κB pathway, activating the TrkB pathway, and maintaining BBB integrity [179]. Catalpol enhances memory by upregulating BDNF expression and protects forebrain neurons from neurodegenerative disorders [180]. There was a strong correlation between synaptophysin and GAP-43 and the rise in PKC and BDNF in the hippocampal region of the catalpol-treated group when compared to rats. Research findings suggested that catalpol could upregulate the pertinent signaling molecules and raise the levels of presynaptic protein in the hippocampal regions of aged rats. Therefore, by “normalizing” presynaptic proteins and the signaling pathways that are linked to them in aging rats, catalpol may help to mitigate age-related neuroplasticity loss [181]. By preventing apoptosis, catalpol can also dramatically raise the brain’s BDNF levels, which boosts the survival rate of immature neurons [182]. A recent investigation found that TrkB-mediated synaptic plasticity is important in the antidepressant characteristics of catalpol and that inhibiting COX-2 is likely a necessary facilitator of catalpol’s antidepressant efficacy via the TrkB target and TrkB-mediated synaptic plasticity [183].

### 6.4. Alkaloids

Sesquiterpene alkaloid huperzine A, derived from the *Huperzia serrata* plant, provides neurological protection by suppressing acetylcholine-esterase (AChE) activity, modifying Aβ peptide processing, and promoting antiapoptotic protein expression and NGF [184]. According to some studies, huperzine A treatment in SH-SY5Y neuroblastoma cells raise the NGF level brought on by H_2_O_2_-induced oxidative stress [185]. This effect was enabled by activating the p75NTR and TrkA receptors and the upstream MAP/ERK signaling pathway. Furthermore, in rat PC12 cells and rat cortical astrocyte cells, huperzine A stimulates neuron outgrowth by reducing AChE and enhancing the expression of NGF and p75NTR [186]. Another alkaloid, berberine, extracted from the *Coptis chinensis*, carries a variety of neuroprotective properties. It has been seen in rats with scopolamine-induced memory deficits that berberine was found to rapidly decrease the secretion of the pro-inflammatory cytokines Cox-2, IL-1β, and TNF-α and significantly increase levels of BDNF and CREB while also shortening the escape latency [187]. In another experiment, berberine pretreatment reduced the expression of Cox-2 and iNOS in BV2 and primary microglia cells and prevented Aβ from inducing the production of IL-6 and MCP-1. This was accomplished via phosphorylating IκB-α and NF-κB through the activation of AKT/ERK1/2 [188].

Natural nicotine produced by *Nicotiana tabacum* has been shown to increase BDNF levels and ameliorate chronic memory dysfunctions brought about by the buildup of Aβ [189]. Nicotinic acetylcholine receptor (7nAChR)-mediated mechanisms support nicotine to protect against AD. Through the 7nAChR, nicotine reduces the clustering of Aβ in the hippocampus and cortex of amyloid precursor protein (V717I) transgenic mice [190]. At the cellular level, nicotine triggers the α7-nAChR/Elk signaling pathway to improve damaged mitochondrial membrane potential, prevent damage to hippocampus neurons from the excessive accumulation of reactive oxygen species (ROS), and lessen oxidative damage from hydrogen peroxide [191]. By inhibiting the activity of MAPK, which results in the activation of iNOS and the down-regulated synthesis of nitric oxide, nicotine prevents the expression of NF-κβ and c-Myc oncogene [192].

### 6.5. Others

The bark extract of *Eucommia ulmoides*, rich in iridoid glucosides (geniposidic acid), prevents NDs such as AD by regulating the expression of fragmented poly(ADP ribose) polymerase (PARP), fragmented caspase 3, Bcl-2, and Bcl-xL through blocking JNK, p38 MAPK, ERK1/2, and PI3K/AKT signaling in H_2_O_2_ treated human SH-SY5Y neuroblastoma cells [156]. In an earlier study, it was found that geniposidic acid efficiently enhanced BDNF expression and decreased AChE activity by boosting cholinergic signaling in scopolamine-induced memory deficit mice. Additionally, *E. ulmoides* bark extract prevents Aβ25-35-induced cognitive impairments in the brain [157]. 

The *Magnolia officinalis* lignin components honokiol and magnolol significantly reduce ROS production, diminish intracellular calcium levels, and suppress caspase 3 to prevent Aβ-induced neuronal cell death. They also trigger PC12 cells’ NGF-mediated differentiation [193,194]. 4-*O*-methylhonokiol, a new lignin compound, enhances ERK1/2-mediated neurite outgrowth in rat embryonic neural cells by stimulating NGF and BDNF secretion [195]. Moreover, magnolol raises the levels of BDNF expression in the serotonergic system in the brains of rats who have experienced unpredictable chronic mild stress [196]. Additionally, honokiol therapy increased the production of cell cycle-related proteins such as cyclin D1, CDK4, pRb, and E2F1 to counteract the neuronal death and dysfunction brought on by traumatic brain injury-induced apoptosis [134].

## 7. Synergistic Effects and Combination Therapies

Scientists discovered that substances including baicalein, tanshinone IIa, cinnamic acid, epiberberine, genistein, and wogonin were among the natural compounds that increased BDNF release from cultured rat primary cortical neurons in an initial screening. BDNF release was elevated among numerous other factors. At a 0.1 μM concentration, none of the compounds significantly stimulated BDNF induction; however, their combination (mixture 1: baicalein, tanshinone IIa, and cinnamon; mixture 2: epiberberine, genistein, and wogonin) demonstrated a synergistic increase in mRNA and protein expression and BDNF release. Combining natural substances on rat primary cortical neurons resulted in synergistic stimulation of BDNF synthesis release [197]. Incorporating combinations of compounds as therapeutic agents offers various benefits over employing a single molecule in higher concentrations [198]. Many plant-derived bioactive compounds have been shown to neutralize ROS and enhance cellular antioxidant systems. The latter method of producing an antioxidant effect by increasing the adaptive cellular stress response using phytochemicals is well supported [199]. 3,6′-Disinapoyl sucrose (DISS) and tenuifoliside A (TFSA), two naturally occurring oligosaccharide esters, are derived from the root of Polygala tenuifolia Willd, a plant used in traditional Chinese medicine to treat mental disorders. According to earlier studies, they both have neuroprotective effects in vitro through activating distinct upstream pathways associated with cyclic AMP-responsive element-binding protein (CREB). Biochemical studies showed that DISS and TFSA reduced Glu-induced NOS hyperactivation while upregulating CREB phosphorylation and CRTC1 and BDNF expression [200]. The combination of the six-compound combination (SCC) and Banxia–Houpo decoction improved depressive behaviors in corticosterone-lesioned mice, protected HD PC12 cells, and induced 5-HT1A receptor expression in mice and BV2 microglia. The antidepressant effects were reduced by the 5-HT1A antagonist WAY-100635, suggesting the combination’s potential in treating depression and neurological disorders through 5-HT1A modulation in microglia [201]. Another study showed the combination of geniposide and shanzhiside methyl ester elevated PACAP expression in the hippocampus, resulting in increased BDNF signaling via CaMKII and mTOR pathways. This combination significantly reduced depression-like behaviors, demonstrating its potential as a quick-acting antidepressant by altering hippocampus BDNF expression [202].

It has been demonstrated that giving BDNF directly to the rat brain improves learning and memory in animals with cognitive impairment [203]. Unfortunately, due to its low bioavailability, its incapacity to cross the blood–brain barrier, and the negative side effects that have been reported its from oral administration, BDNF is not a viable therapy choice [204]. Alternative methods of stimulating TrkB with small-molecule activators have been investigated [205]. Although resveratrol, a phytochemical that targets BDNF, has demonstrated promise in human trials, there are major barriers to its clinical application. Its limited bioavailability upon oral administration is the primary problem, as it restricts its effectiveness. Comparing results is made more difficult by the substantial heterogeneity and inconsistent methodologies found in a meta-analysis of clinical research. Furthermore, trustworthy conclusions are further hampered by the absence of established protocols in these experiments. To increase bioavailability and guarantee therapeutic efficacy, future studies should concentrate on standardizing trial designs, employing optimal formulations, and determining appropriate dosages (100–500 mg) [206].

## 8. Challenges and Limitations in the Therapeutic Application of Phytochemicals

Many phytochemicals, including resveratrol and ginkgolic acids, have low oral bioavailability due to their low water solubility and fast metabolisms. Despite its high potential as a therapeutic agent for a variety of disorders, RES has certain limitations. It has low water solubility and is chemically unstable, degrading via isomerization when subjected to high temperatures, pH changes, UV light, or certain enzymes. Thus, RES has low bioavailability, which limits its biological and pharmacological advantages. To overcome these constraints, RES can be given by nanocarriers [207].

Curcumin, and flavonoids such as quercetin, *Ginkgo biloba*, and folic acid have sparked the interest of scientists, and methods are being developed to boost their efficacy and bioavailability at the site of action. Most compounds have challenges with solubility, bioavailability, or the ability to penetrate the blood–brain barrier, and nanomedicine is one option being investigated to address this constraint. Polymeric nanoparticles, liposomes, and functionalized nanosystems may overcome many bioavailability barriers to active compounds and boost their efficacy in the brain [208]. Many of these compounds are poorly soluble in water, have low bioavailability, and undergo substantial first-pass metabolism in the gastrointestinal and/or hepatic systems, resulting in rapid elimination and low serum and tissue concentrations. Thus, the intranasal route has emerged as a feasible alternative to oral or parenteral administration, allowing for direct delivery into the brain via the olfactory and trigeminal nerves. This technique bypasses the blood–brain barrier and reduces peripheral exposure, hence reducing the possibility of unwanted consequences [209].

## 9. Current Therapeutic Limitations

Many synthetic drugs are being used to treat NDs; however, because of their adverse effects, these drugs are having major negative reactions generated by a medicine that has an influence on a patient’s health, quality of life, or treatment outcomes [210]. These consequences are usually more severe than mild side effects and may necessitate medical intervention, dose modifications, or withdrawal of the medicine.

Based on a literature review, drug discovery against the neurological disorder is deemed insufficient, with only a few clinically approved treatments with low efficacy. Natural products are garnering interest as treatment options due to their molecular variation, safety, and efficacy. Phytochemicals, particularly phenolic compounds, have been shown to improve neurodegeneration caused by oxidative stress, lower Aβ levels, and inhibit key enzymes [211]. However, their clinical efficacy and bioavailability remain as major issues, necessitating further treatments for better therapeutic outcomes [211]. These natural compounds have antioxidant, anti-inflammatory, and angiogenic activity, protect the neurovascular unit and blood–brain barrier integrity, and influence neural stem cell differentiation, synapse formation, and neurogenesis, making them potential therapeutics for neurological disorders. These natural substances have been demonstrated in different in vivo and in vitro research, as well as clinical trials, to be safe and acceptable, with few side effects [212]. A number of plant components have been found to show potent therapeutic properties against a range of neurodegenerative illnesses by neurotrophin regulation. Several earlier studies revealed that frequent consumption of phytochemicals improved mental and physical function, increased neuronal cell life, and strengthened the antioxidant system [213]. Therefore, the objective of the present review is to provide in-depth knowledge of phytochemicals targeting the neurotrophins, specifically BDNF, for the treatment of NDs.

## 10. Future Directions for Targeting BDNF Signaling

Despite its role in fundamental physiology being well established, BDNF investigations can deliver contradictory and ambiguous results, and the amount of remaining research that needs to be carried out makes determining future directions difficult. Enhancing therapies and patient outcomes can be achieved by comprehending the basic physiology of when, where, and how BDNF works as well as novel strategies to regulate BDNF signaling in different situations [214]. Physical and chemical instability, quick metabolism, BBB crossing, low bioavailability, and decreased water solubility are some of the issues and limitations that natural products and isolated chemical compounds come with and can affect their therapeutic efficacy [215].

## 11. Conclusions

NDs are a complicated and wide-ranging category of illnesses that impact the nervous system. These are the largest causes of death and disability worldwide and are receiving more attention as laboratory research is transferred into clinical practice. The physiological and functional disruptions of neurons constitute the defining feature of these disorders. Numerous phytochemicals, such as phenols, steroids, and terpenoids, have been shown to have neuroprotective effects that can prevent and reduce the progression of NDs. These compounds are also economical, simple to apply, and easily available, as well as show promise for applications in the future.

A natural compound that influences the nervous and peripheral systems of the brain, boosting cognition, mood, and brain function, is generated from herbs, spices, and herbal products and performs better than other substances in terms of energy production, brain biogenesis, and neuroprotection. Plant extracts’ chemical profiles must be determined through proper characterization and standardized extraction processes. Dosage constancy is required for standardized outcomes. Long-term safety and toxicity should be assessed.

According to the aforementioned analysis, the first-line treatment for several NDs should be the phytochemicals that target upstream BDNF neurotrophins and other signaling targets downstream, as NGF only promotes expansion and the existence of growing neurons. Although they might not be a complete panacea, phytochemicals that stimulate BDNF may help delay or prevent the onset of NDs. Furthermore, phytochemicals do not seem to be cytotoxic according to their chemical profile. Additionally, they enable neurons to renew and maintain mature neurons in a suitable environment.

Although the phytochemicals with NGF potential properties are beneficial in in vitro neuron cell models, several challenges need to be solved before clinical trials. To demonstrate the impact of phytochemicals in preliminary in vivo models, additional research should be performed. In-depth research is particularly required to clarify which phytochemicals influence neurodegenerative illnesses by controlling NGF-TrkA signaling. In conclusion, this review has discussed the neuroprotective properties as well as the molecular mechanisms of phytochemicals through which they work against NDs.

## Figures and Tables

**Table 1:** Phytochemicals extracted from plants with activities and the mechanism involved in therapy.

Phytochemicals	Name of the Plant	Mode of Action	Disease Pathology	Doses	References
*Steroids*					
Spicatoside A	*Liriope platyphylla*	Enables Trk, ERK1/2/PI3K-mediated neurite development, and stimulates the release of NGF and BDNF.	Alzheimer’s disease (AD)	10 μg/mL, 2.5–20 mg/kg.	[138,139]
*Phenols*					
Apigenin-8-C-β-digitoxopyranoside, apigenin-8-C-β-boivinopyranoside, and luteolin-8-C-β-boivinopyranoside	*Passiflora edulis* (L.)	Inhibits ERK1/2/CREB signaling, reduces BACE, β-CTF, and β-amyloid deposition, and improves BDNF expression.	Alzheimer’s disease (AD)	40 mg/kg for 5 days/week	[140]
Epigallocatechin-3-gallate	*Camellia sinensis*	Reduces Aβ(1–40) expression, enhances NGF and CREB expression by TrkA phosphorylation through c-Raf/ERK1/2-mediated neuroprotection.	Amyotrophic Lateral Sclerosis (ALS)	10 mg/kg body	[141]
Demethoxycurcumin, bisdemethoxycurcumin, and curcumin	*Curcuma longa*	Induces BDNF production, activates PKC/ERK and AKT/GSK-3β-mediated CREB regulation, and reduces Cas3, TNF-α, and NF-κB levels.	Alzheimer’s disease (AD)	5 μg/mL/30 min	[142]
Resveratrol	*Vitis vinifera*	Raises SOD levels, promotes ERK-mediated CREB regulation, stimulates the production of NGF, GDNF, and BDNF, and inhibits the activity of caspase 3, TNF, NF-κB, IL-10, IL-1, MCP-1, and MDA.	Alzheimer’s disease (AD)	16 mg/kg/day	[143]
Silibinin	*Silybum* *marianum*	Increases BDNF and TrkB expression and enhances autophagy.	Alzheimer’s disease (AD)	25, 50, and 100 mg/kg for 10 days	[144]
Rutin	Many Plants	Increases BDNF secretion, reduces ROS and NO, caspase 3 activity, caspase 8, FAS, and FASL.	Prion disease pathology	10 μg/mL	[145]
6-Shogaol	*Zingiber* *officinale*	Initiates Trk-mediated neuronal development, enhances GDNF, BDNF, and NGF production, and prevents Cox2, TNF-α, NF-κB, IL-1β, NO, p38, iNOS, Bax, PG-E2, and ROS levels.	Neural apoptosis and Alzheimer’s disease (AD)	10 μM	[146,147]
Oleuropein	*Olea europaea*	Enhances NGF and BDNF secretion and GSH levels.	Disease pathology is not explicitly mentioned.	20 mg/kg	[148]
*Terpenoids*					
Ginkgolide B	*Ginkgo biloba* (L)	Suppresses ROS, LDH, caspase 3, and pro-apoptotic proteins; activates Trk/Ras/MAPK-mediated neurite propagation; and induces BDNF secretion.	Alzheimer’s disease (AD)	100 mg/kg for 1 month	[149]
3,4-secocycloartene triterpenoid, α-Iso-cubebene, dibenzocyclooctadiene lignans, schisanchinins A–D	*Schisandra chinensis*	Activates the Ca^2+^-calmodulin-mediated kinase II and ERK1/2 signaling pathways, and inhibits NO generation, and induces BDNF and c-Fos expression.	Parkinson’s disease (PD)	(1, 10, 50 μM)	[150,151]
Catalpol	*Radix preparata*	Upregulate cyclin-dependent kinase 5 (Cdk5), several regulatory proteins (BDNF), and p35.	Attention Deficit Hyperactivity Disorder (ADHD).	50 mg/kg/day	[152]
*Alkaloid*					
Huperzine A	*Huperzia* *serrata*	Suppresses AChE activity, modifies Aβ peptide processing, activates the p75^NTR^ and TrkA receptors and the upstream MAP/ERK signaling pathway, and enhances MMSE, CDR, and ADL scores.	Alzheimer’s disease (AD), vascular dementia (VaD)	0.1 mg bid	[153,154]
Nicotine	*Nicotiana* *tabacum*	Significantly decreases growth factor (NGF) and brain-derived neurotrophic factor (BDNF) in the frontal cortex and hippocampal regions of neonatal rats.	mechanisms underlying a range of conditions like ADHD, depression,	66 μg	[155]
*Others*					
Geniposidic acid	*Eucommia* *ulmoides*	Regulates PARP, caspase 3, Bcl-2, and Bcl-xL expression; inhibits JNK, p38 MAPK, ERK1/2, and PI3K/AKT signaling; enhances BDNF expression; reduces AChE activity.	Alzheimer’s disease (AD)	(5, 10, or 20 mg/kg, p.o.)	[156,157]
Honokiol, magnolol	*Magnolia* *officinalis*	Enhances Akt activity and NGF and BDNF secretion, and reduces TNF-α, NF-κB, IL-1β, IL-6, and ROS levels.	ischemic stroke	0.7–70 μg/kg, 15 min after ischemia	[158]

**Table 2:** The table shows clinical trials utilizing certain phytochemicals, as acquired from ClinicalTrials.gov. The table includes the compound name, study title, target condition, trial status, NCT number, and intervention information. These investigations looked into the therapeutic potential of natural substances in a variety of ailments, including neurological problems, metabolic and inflammatory disorders. The table shows completed trials, indicating clinical interest in certain phytochemicals for potential medical applications.

Compound Name	Study Title	Condition	Status	NCT Number	Intervention
honokiol magnolia	A Clinical Study to Evaluate the Efficacy of Dietary Supplement to Alleviate Stress Versus Placebo in Subjects with Mild to Moderate Levels of Stress	Evolutions, Self-Diagnosis	Completed	NCT06672965	Dietary Supplement: Anti-Stress Dietary Supplement
*Curcumin*	Curcumin Gel On Radiation Induced Oral Mucositis	Radiation-Induced Mucositis Head and Neck Cancer	Completed	NCT05982197	Drug: Curcumin Gel Drug: Standard Preparation
*Curcumin*	Micro-Particle Curcumin for the Treatment of Chronic Kidney Disease	Chronic Kidney Disease	Completed	NCT02369549	Drug: Micro-Particle Curcumin Drug: Placebo
*Eucommia ulmoides*	Efficacy and Safety of Cortex Eucommiae (CE: Eucommia Ulmoides Oliver Extract) in Subjects With Mild Osteoarthritis	Osteoarthritis	Completed	NCT03744611	Dietary Supplement: CE Dietary Supplement: Placebo
*Schisandra chinensis*	A Study With Arctic Root Compared With the Extract When Combined With Schizandra and Russian Root (Adapt 232), Standa0rdized Ginseng Extract and Placebo Regarding Impact on the Level of Energy, Ability to Work Under Stress, Quality of Life and Wellbeing, in Middleaged Women Who Are Still Employed	Depression Stress	Completed	NCT01006460	Drug: *Rhodiola rosea*, L Drug: *Rhodiola rosea*, L., *Eleutherococcus senticosus*, *Schisandra chinensis* Drug: *Panax ginseng*
*Schisandra chinensis*	Body Constitution Classification Based Comprehensive Health Management Intervention on Obese Population	Overweight Obesity	Completed	NCT02298426	Behavioral: Health Management Behavioral: General Management Other: Placebo
*Camellia sinensis*	Effect of Quercetin on Green Tea Polyphenol Uptake in Prostate Tissue From Patients With Prostate Cancer Undergoing Surgery	Adenocarcinoma of the Prostate Recurrent Prostate Cancer Stage I Prostate Cancer	Completed	NCT01912820	Dietary Supplement: green tea extract Drug: quercetin Other: placebo
*Camellia sinensis*	Mobile Mental Health Apps for Suicide Prevention	Depression Anxiety Emotional Regulation	Completed	NCT04536935	Behavioral: Mobile Mental Health App—1 Behavioral: Mobile Mental Health App—2 Behavioral: Mobile Mental Health App—3
